# High Fat and Sugar Diet Increases Enteric cDC1 and Oral Antigen‐Specific Tregs

**DOI:** 10.1155/jimr/6600076

**Published:** 2025-10-28

**Authors:** K. Fischer, O. Horno, B. Raposo, I. Godinho, H. Ribeiro, H. Veiga-Fernandes, M. Martínez-López

**Affiliations:** ^1^ Immunophysiology Group, Champalimaud Centre for the Unknown, Champalimaud Research, Champalimaud Foundation, Lisbon, Portugal, fchampalimaud.org; ^2^ Faculty of Science and Technology, Norwegian University of Life Sciences (NMBU), N-1432, Ås, Norway, nmbu.no; ^3^ Champalimaud Neuroscience Programme, Champalimaud Centre for the Unknown, Champalimaud Research, Champalimaud Foundation, Lisbon, Portugal, fchampalimaud.org

## Abstract

Diet‐induced obesity is a growing global health concern linked to various immunological alterations. Dendritic cells (DCs) are major regulators of the balance between pro‐inflammatory and tolerogenic immune responses. Conventional Type 1 DCs (cDC1) contribute to oral tolerance by affecting the generation of food specific regulatory T cells (Tregs). Nevertheless, whether obesity affects cDC1/Treg pathways and the generation of food tolerance remains poorly understood. Here, we investigated the spatio‐temporal impact of a high‐fat, high‐sugar diet (HFHSD) on the enteric immune system. Enteric immune composition was primarily altered by diet and intestinal region, independently of the duration of the dietary regimen. Notably, the lamina propria of animals fed with high‐caloric diet was enriched in cDC1 overtime. While diet did neither reprogramme cDC1 gut‐homing markers, nor costimulatory molecules nor cytokines, it increased intestinal cDC1 levels, which correlated with increased Tregs during oral tolerance protocols. Our findings contribute to a better understanding of high‐caloric diet and food intolerances, while revealing a remarkable plasticity of the intestinal immune system in response to diet.

## 1. Introduction

Obesity has become a growing global health concern. Obesity‐associated chronic, low‐grade inflammation affects various organs including the intestine [1]. Prevalence for food intolerances is increasing, and emerging evidence suggests a potential link between dietary lipid intake and oral tolerance [2].

Conventional dendritic cells (cDCs) are key regulators of metabolic and immune homeostasis, acting as antigen‐presenting cells that initiate adaptive immune responses. cDCs arise from common DC progenitors (CDPs) in a process highly influenced by the tyrosine kinase 3 ligand (FMS‐like tyrosine kinase 3 ligand [Flt3L]), amongst others [3]. cDC1 and cDC2 subsets have distinct markers, functions and developmental pathways. In brief, cDC1s express XCR1, CD103 and DNGR‐1, whereas cDC2s mainly express SIRP1α and CD11b. cDC1s are essential to initiate CD8^+^ cytotoxic T lymphocyte (CTL) response against viruses and tumours, whereas their contribution to CD4^+^ T cells activation is less clear. In contrast, cDC2 are fundamental in inducing CD4^+^ Th2 and Th17 responses [4].

A particularly interesting immunological site of the body is the small intestine, where tolerance to food antigens and commensals and the ability to respond to pathogens coexist [5].

cDCs sample antigens in the intestinal lumen, and early data indicated that cDC1s are essential initiators of food specific peripheral regulatory T cells (pTregs) responses by migrating to mesenteric lymph nodes (mLNs) in a CCR7‐dependent manner and producing TGF‐β, retinoic acid and tryptophan metabolites [6–8]. Under inflammatory conditions, changes in mLN cDC1/cDC2 frequencies were also shown to affect oral tolerance [9]. More recently, it was shown that RORγt^+^ antigen presenting cells (APCs) contribute to the induction of food‐specific pTregs. RORγt^+^ APCs possibly play a dominant role in the generation of pTregs, while cDC1s likely sustain pTreg cells and suppres CD8^+^ T cell responses [10–14].

Obesogenic diet changes cDC representation in a tissue‐specific manner: bone marrow (BM) DC progenitors and cDC1s in the small intestine increase, while cDCs in adipose tissue decrease [1, 15, 16]. Moreover, cDCs of the obese host switch to a more anti‐inflammatory phenotype that could facilitate Treg induction [1]. Our study demonstrates that the lamina propria of obese animals is enriched in cDC1s displaying normal gut‐homing markers, costimulatory molecules and cytokines. Increased intestinal cDC1s correlated with increased Tregs upon food antigen challenge, suggesting enhanced oral tolerance during high‐caloric diet regimens.

## 2. Results

### 2.1. Obesogenic Diet Reshapes the Host Enteric Immune System

To investigate the impact of an obesogenic diet on the enteric immune system, C57BL/6Jl mice were fed either a control diet (CD) or a high‐fat, high‐sugar diet (HFHSD). As expected, 4 weeks after dietary intervention, HFHSD‐fed mice had increased body weight and decreased tolerance to glucose compared to CD‐fed animals (Supporting Information 1: Figure S1a,b). Duodenal, jejunal and ileal lamina propria and intra‐epithelium immune compartments were analysed by flow cytometry at 4, 8 and 16 weeks of dietary intervention (Figure 1a and Supporting Information 1: Figure S1c).

Figure 1The adult enteric immunophenotype is dependent on the diet of the host and intestinal segment. (a) Experimental setup. Male C57BL/6 mice were fed CD until 8 weeks of age. Mice were then either fed HFHSD or CD. Immune compartment of lamina propria and epithelium of duodenum, jejunum and ileum was analysed at indicated time points by flow cytometry. (b, c) PCA representation of enteric immunophenotypes (*n* = 15). Each data point represents the cluster of the immune landscape of one intestinal segment at one time point in one condition, CD or HFHSD. Data points are coloured by (b) diet and experimental time point or (c) diet and intestinal segment. Data are pooled from three independent experiments. *n* represents biologically independent animals.

**Figure:**
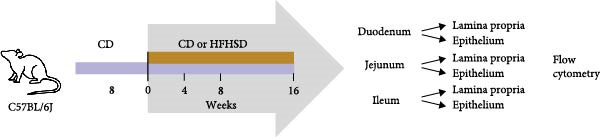
(a)

**Figure:**
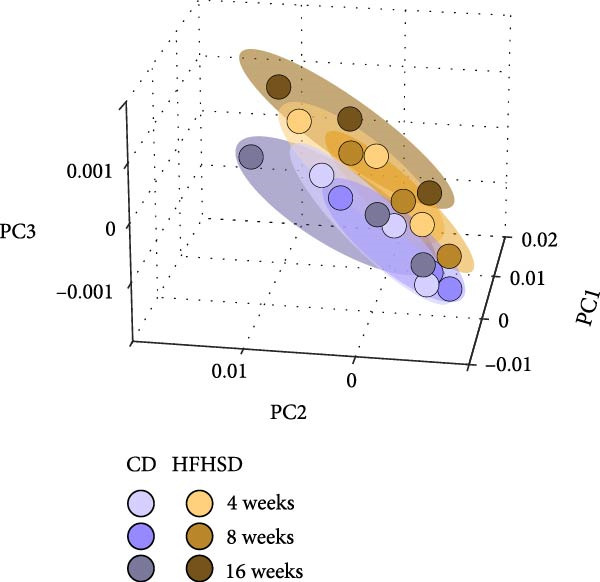
(b)

**Figure:**
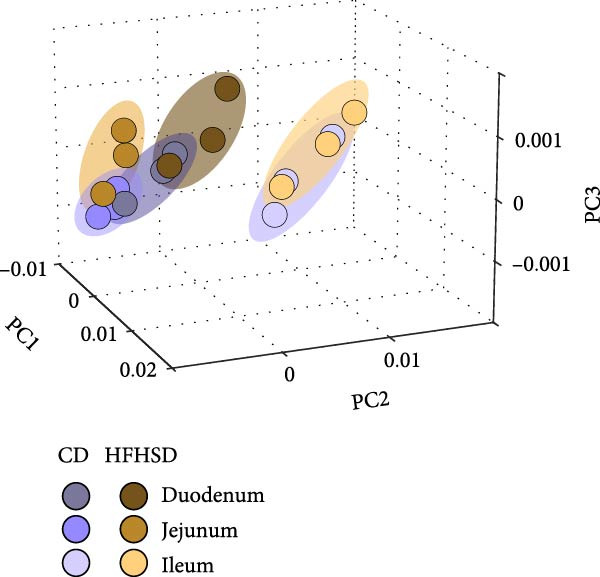
(c)

To assay the impact of diet, duration of dietary intervention, age of the animal and intestinal segment on the enteric immune composition, we employed principal component analysis (PCA). We observed distinct clustering patterns between HFHSD and CD groups, indicating substantial alterations in the composition of the immune system due to dietary intervention. Age or duration of specific diet did not segregate clusters (Figure 1b). However, there were different clusters dependent on intestinal segments (duodenum, jejunum or ileum), while the same enteric segments of different diets clustered together (Figure 1c). Moreover, data from ileum showed greater distance to data from duodenum and jejunum, indicating greater similarities between the immune composition of the proximal and middle part of the small intestine and a clear distinction between those and the distal small intestine. These initial findings identified diet and intestinal location as the primary drivers of the enteric immune composition.

### 2.2. Enteric cDC1 Increase Upon Obesity

To explore the impact of diet and intestinal location on the enteric immune composition, we interrogated the effect of these factors on specific intestinal immune cell types. The effect of experimental time point, dietary intervention and duration of intervention on the composition of the intestinal immune system was assessed by two‐way repeated measures ANOVA between individuals. The effect of dietary intervention and intestinal segment was assessed by two‐way mixed repeated measures ANOVA that compares within and between individuals. Regardless of the intestinal segment or time point, dietary intervention alone was shown to have a significant impact on the ratio of total cDC, B cells and CD8^+^ T cells in the lamina propria (Figure 2a). In the intra‐epithelial compartment, only CD8αβ^+^ T cells were significantly affected by diet alone. Post hoc analysis revealed a consistent effect of diet on different intestinal segments. Likewise to the consistent effect of diet on segments, longer exposure to diet did not invert the effect of diet. Of all the immune cells that were affected by diet, cDC1s were the most consistently affected. cDC1 frequency was increased upon HFHSD from 4 to 8 and from 8 to 16 weeks in both jejunum and ileum lamina propria (Figure 2b and Supporting Information 2: Figure S2a).

Figure 2Kinetics of effect of diet and location on immunophenotype. (a) Effect of diet, time point, intestinal segment and interaction of these factors on frequencies of selected lamina propria and intraepithelial immune cell types. Row 1–3: Two‐way repeated measures ANOVA for tests between subjects (diet and time point). Row 4–6: Post hoc pairwise comparison with Bonferroni correction. Row 7–9: Mix‐repeated measures ANOVA for tests between and within subjects (diet and intestinal segments). Row 10–12: Post hoc pairwise comparison with Bonferroni correction.  ^∗^ *p* < 0.05;  ^∗∗^ *p* < 0.01;  ^∗∗∗^ *p* < 0.005;  ^∗∗∗∗^ *p* < 0.001. E, epithelium; ns, not significant. (b) Ratios of cDC subsets in CD45^+^ cells at indicated time points and intestinal segments in HFHSD or CD conditions (*n* = 15). Data are pooled from three independent experiments. *n* represents biologically independent animals. Data are presented as mean ± s.e.m.

**Figure:**
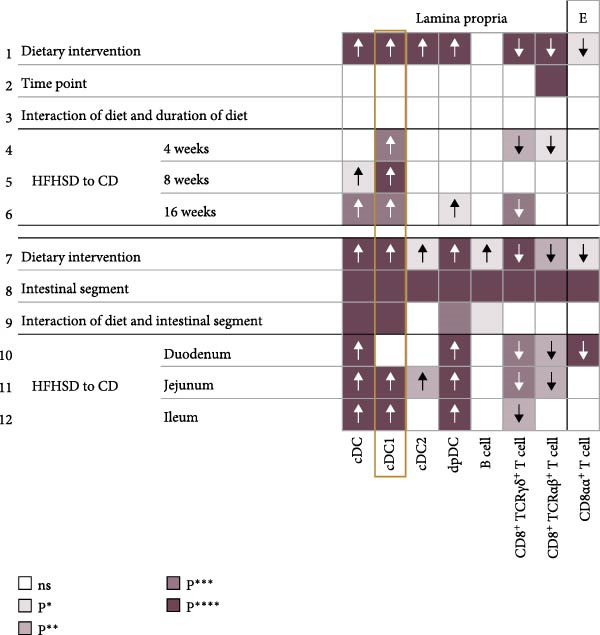
(a)

**Figure:**
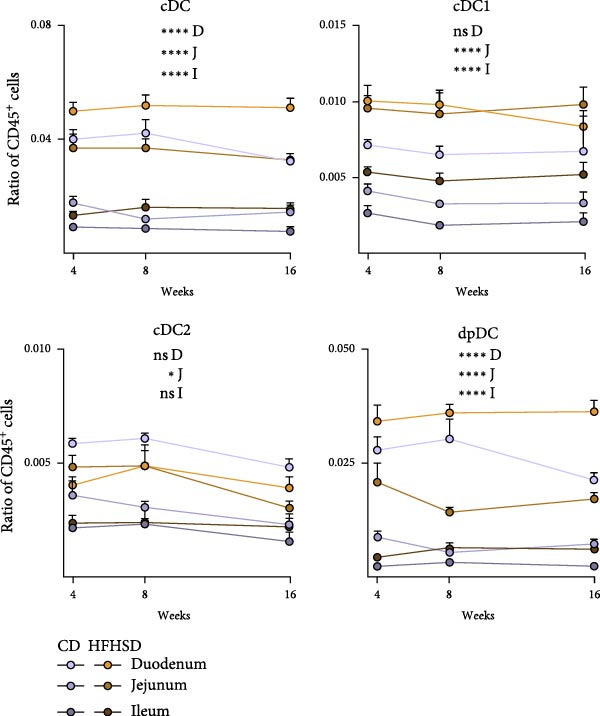
(b)

### 2.3. cDC1 Ontogeny and Organ Distribution in the Obese Host

To investigate the underlying mechanisms driving the pronounced increase in enteric cDC1 frequency upon HFHSD, we focused on cDC1 progenitors. cDCs originate from BM precursors, CDPs, which progress through a series of developmental stages to achieve full differentiation in peripheral tissues [17]. We observed no significant difference in the percentage and total numbers of neither CDPs nor pre‐cDCs in obese compared to steady‐state mice (Figure 3a and Supporting Information 3: Figure S3a,b). Moreover, frequencies and total numbers of pre‐cDC1s, pre‐cDC2s and uncommitted pre‐cDCs were unaffected by HFHSD (Figure 3a and Supporting Information 3: Figure S3a–c).

Figure 3Similar ontogeny of cDC1 in the obese and steady‐state host. Mice were fed for 8 weeks with HFHSD or CD. (a) cDC precursors in bone marrow as percentages in CD45^+^ cells measured by flow cytometry (*n* = 10). (b) Pre‐cDCs as percentages in CD45^+^ cells in the blood and (c) their expression of gut homing molecules as percentages in pre‐cDC1 and MFI (*n* = 10). (d) Flt3L levels in small intestine, serum and bone marrow in pg per organ and pg per g or mL organ measured by ELISA, normalised to total protein (*n* = 15). (e) cDC1 as percentages of cDCs in metabolically relevant organs and (f) as percentage in CD45^+^ cells in mesenteric lymph nodes (*n* = 12). Flow cytometry (a–c, e, f) and ELISA (d). Data are pooled from three independent experiments. *n* represents biologically independent animals. Data are presented as mean ± s.e.m. Two‐tailed unpaired Student’s *t*‐test (a–f).  ^∗^ *p* < 0.05;  ^∗∗^ *p* < 0.01;  ^∗∗∗^ *p* < 0.005;  ^∗∗∗∗^ *p* < 0.001. ns, not significant.

**Figure:**
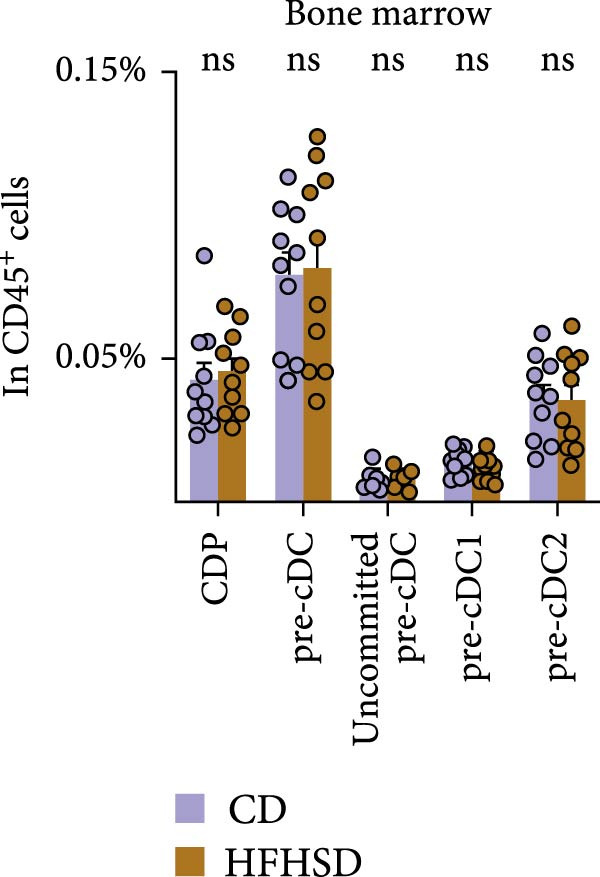
(a)

**Figure:**
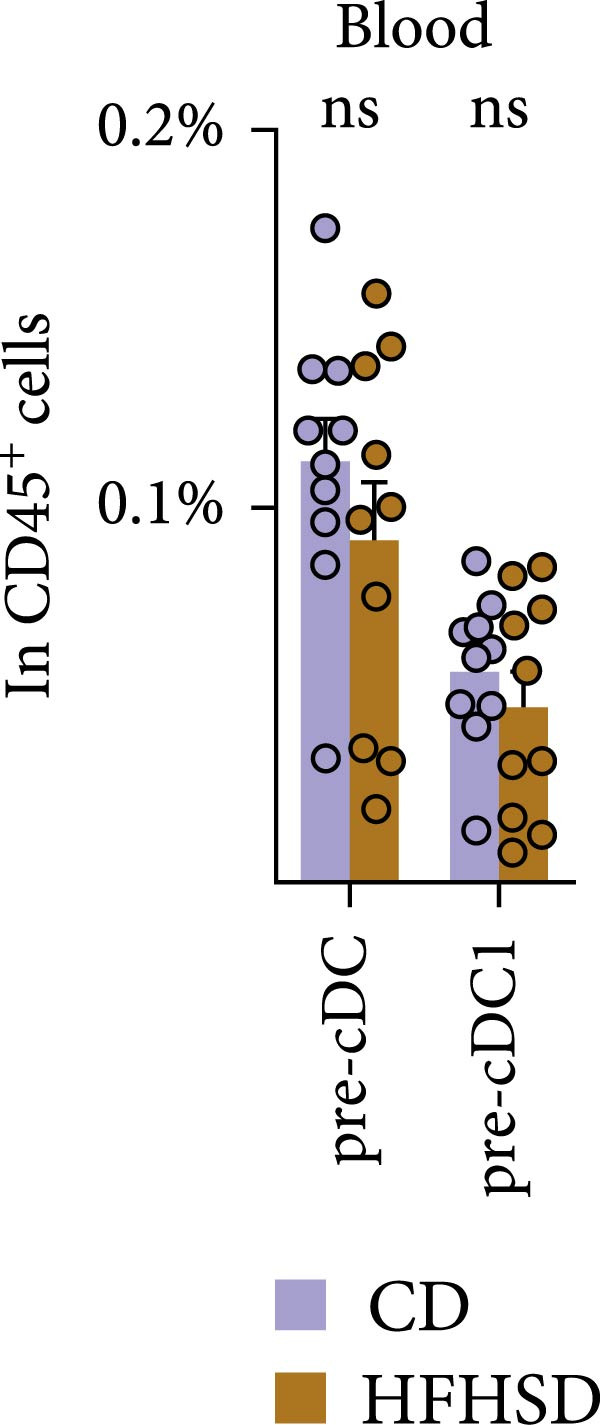
(b)

**Figure:**
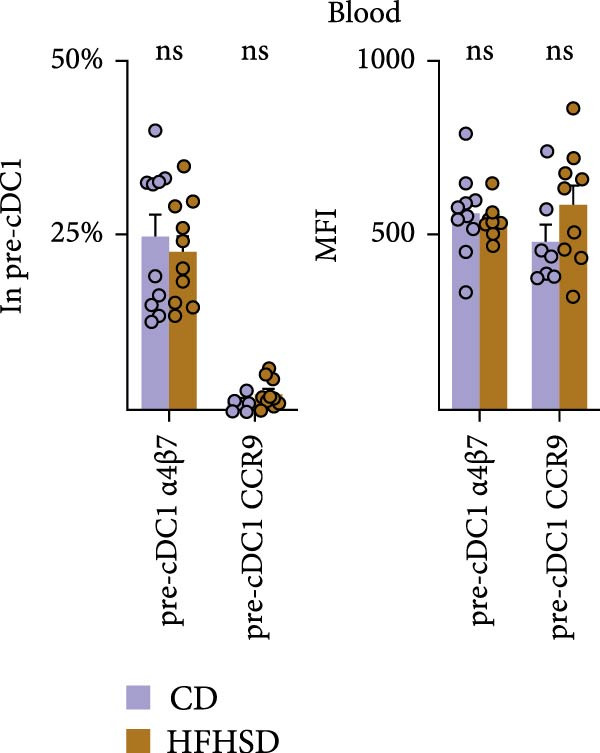
(c)

**Figure:**
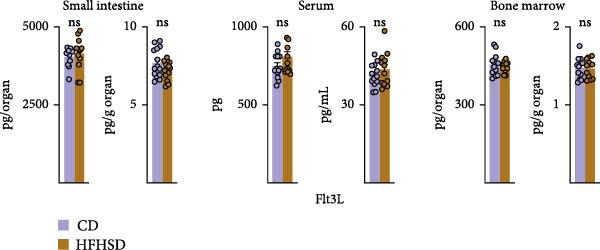
(d)

**Figure:**
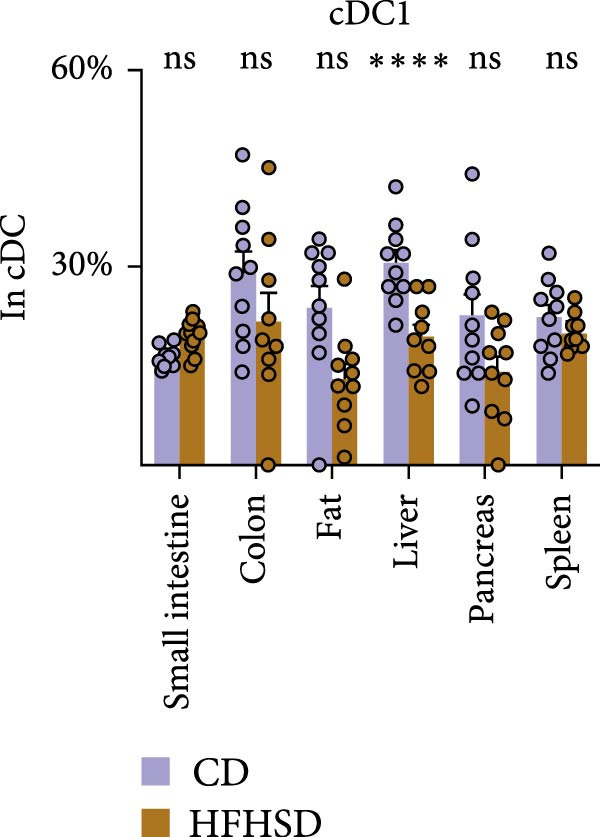
(e)

**Figure:**
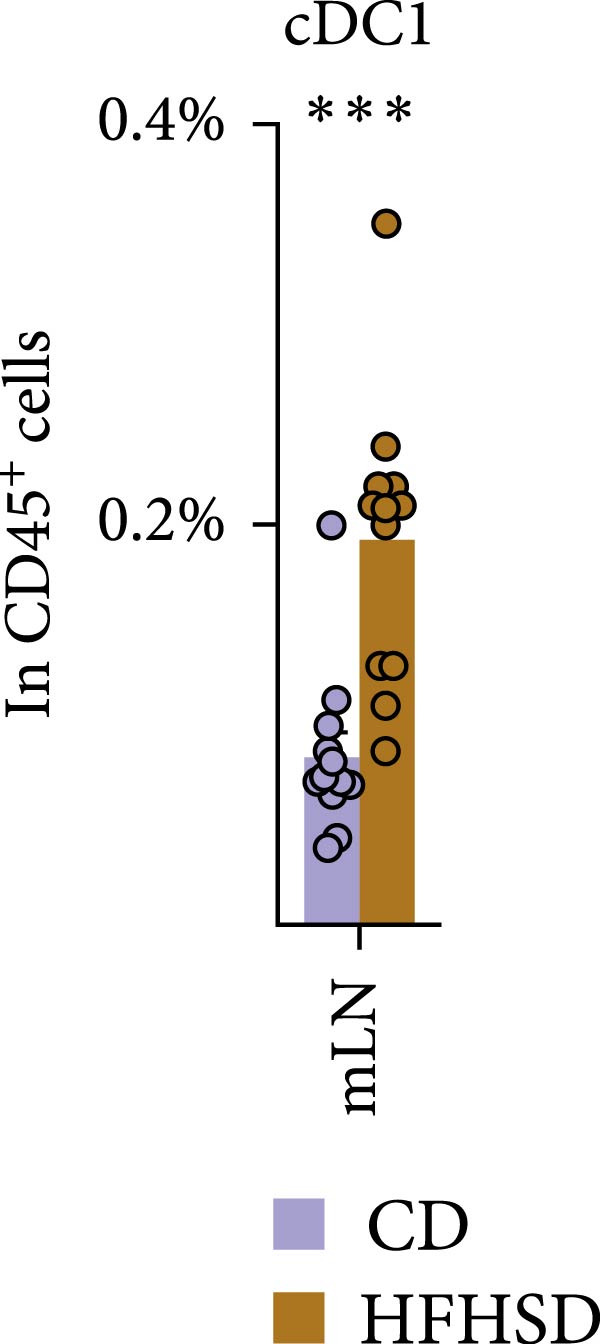
(f)

After BM egression, intestinal cDC homing is induced by the expression of integrin α4β7 and the chemokine receptor CCR9 on pre‐cDCs [18]. To address whether intestinal homing of pre‐cDC1s might have been affected in obesity, these molecules were analysed in pre‐cDC1s. Neither the number of circulating pre‐cDCs nor pre‐cDC1s was significantly altered upon HFHSD (Figure 3b and Supporting Information 3: Figure S3d,e). Likewise, neither percentage of pre‐cDC1s expressing α4β7 or CCR9 nor expression of these molecules per cell was altered upon an obesogenic diet, suggesting no enhanced gut homing capacity of pre‐cDC1s in the obese host (Figure 3c and Supporting Information 3: Figure S3i).

BM cDC development partially relies on the cytokine Flt3L and high Flt3L levels in tissues can locally promote cDC cell division [3]. To test if HFHSD would elevate intestinal Flt3L levels, potentially promoting local cDC proliferation, Flt3L was quantified in the small intestine, serum and BM. Flt3L concentration and total amount did not significantly differ between HFHSD‐ and CD‐fed mice and no enhanced local cDC1 proliferation in the intestine of obese mice was observed, correlating with the steady proportion of pre‐DC precursors in HFHSD settings (Figure 3d; Supporting Information 3: Figure S3f).

We interrogated whether increased intestinal cDC1s upon HFHSD was a result of cDC1s not coming from the BM but rather from other peripheral tissues. To this end, we analysed the frequency of cDC1s in colon, fat, liver, pancreas and spleen. In contrast to the small intestine, all analysed organs showed a tendency for reduced cDC1s in HFHSD‐fed compared to CD‐fed mice, with significant reduction of cDC1s in the liver (Figure 3e and Supporting Information 3: Figure S3g). These data indicate that HSHFD has a differential impact on cDC1 frequency depending on the organ and suggest a potential redistribution of cDC1s between organs upon dietary challenge. In this regard, the observed enteric cDC1 increment in HFHSD could have resulted from different migration capacity, particularly from the small intestine into mLNs. However, we found that cDC1 frequency significantly increased in mLN from HFHSD animals compared to CD animals, suggesting normal cDC1 migration out of the small intestine (Figure 3f and Supporting Information 3: Figure S3h).

### 2.4. Enteric cDC1 Functionality Is Not Altered in Obesity

We tested whether cDC1s maintained their functional properties in the obese state. cDC1 displayed unperturbed expression of co‐stimulatory molecules upon HFHSD (Figure 4a,b). Moreover, when analysing cDC1 expression levels of cytokines by qPCR, we observed a mild downregulation of *Il10* upon HSHFD, suggesting a reduced anti‐inflammatory phenotype of HFHSD cDC1s. However, no differences were observed for *Itgb8* and *Aldh1a* that are essential for the induction of intestinal Tregs (Figure 4c).

Figure 4HFHSD does not alter the status of cDC1 in the small intestine. (a) Flow cytometry gating strategy for cDC1, cDC2 and dpDC. (b) Expression of co‐stimulatory molecules on cDC1 measured by flow cytometry (*n* = 10). (c) Relative gene expression in sorted cDC1 from HFHSD‐fed mice normalised to *Hprt* and CD‐fed mice measured by RT‐qPCR (*n* = 12). (d) Percentage of cDC1 expressing cytokines measured by flow cytometry and expression per cell as MFI (*n* = 12). (e) Histogram showing normalised gene expression of selected genes in cDC1 in scRNA‐seq dataset from Wang et al. [1]. Data are pooled from three independent experiments (b–d). *n* represents biologically independent animals. Data are presented as mean ± s.e.m. Two‐tailed unpaired Student’s *t*‐test (b–d). DESeq2 analysis (e).  ^∗^ *p* < 0.05;  ^∗∗^ *p* < 0.01;  ^∗∗∗^ *p* < 0.005;  ^∗∗∗∗^ *p* < 0.001. ns, not significant.

**Figure:**
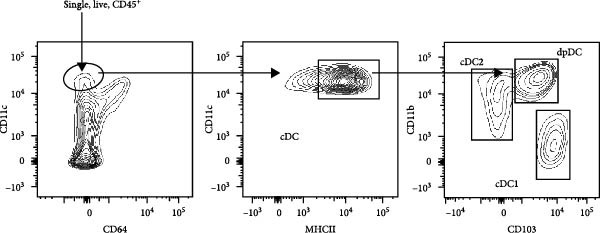
(a)

**Figure:**
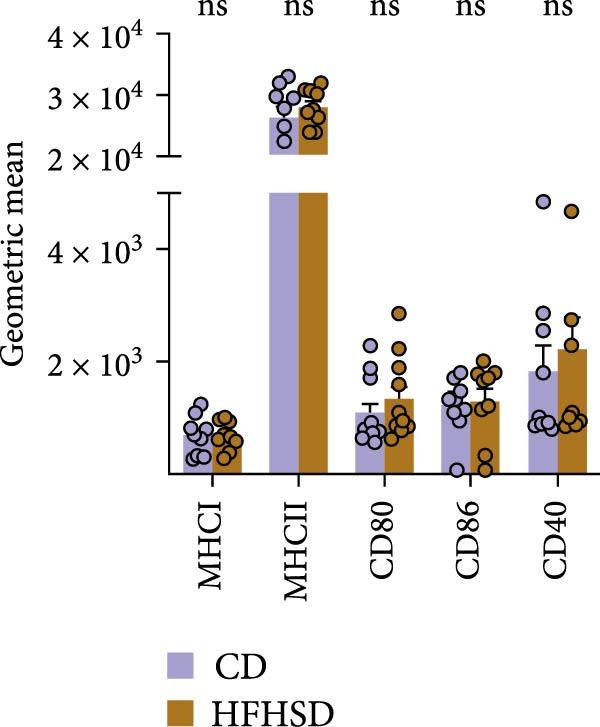
(b)

**Figure:**
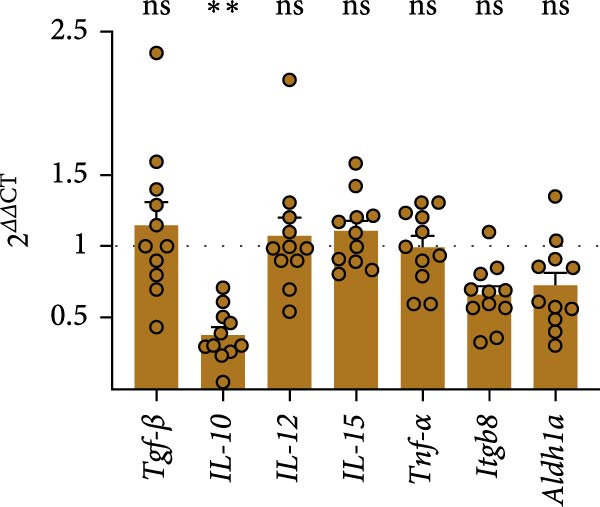
(c)

**Figure:**
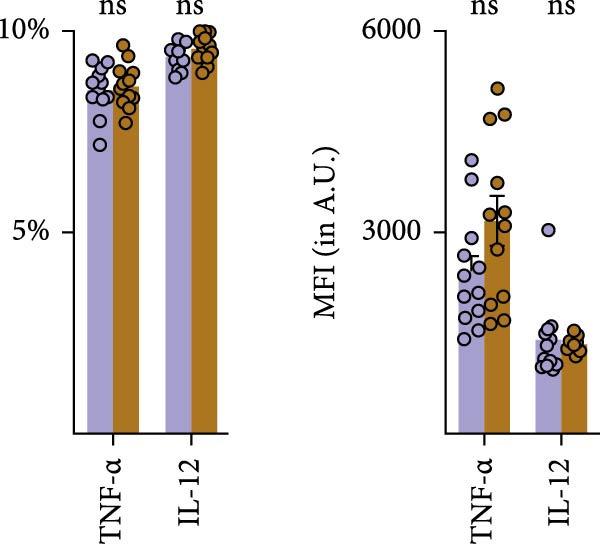
(d)

**Figure:**
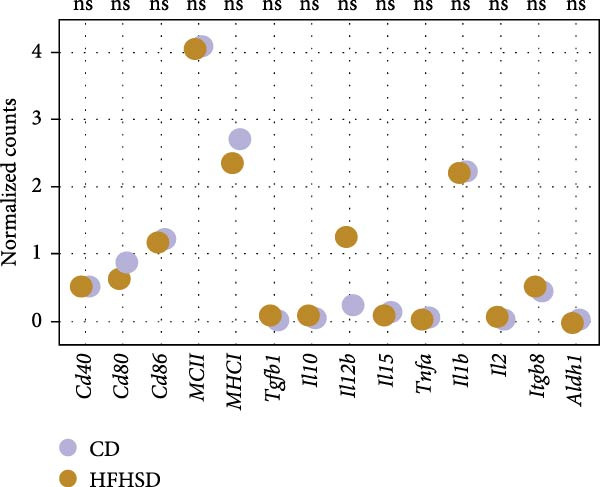
(e)

In line with these findings, enteric cDC1 derived cytokines, notably TNFα and IL‐12, were not altered upon HFHSD at the protein level in vivo (Figure 4c). To further explore cDC1 function in obesity, we analysed publicly available single‐cell sequencing data of the enteric immune system of HFHSD‐ and CD‐fed animals that were comparable to our setup [1] (Supporting Information 4: Figure S4a–c). We identified two clusters inside the DC cluster and confirmed their identity as cDC1 and cDC2 dependent on top differentially expressed genes (DEGs; Supporting Information 4: Figure S4d,e). We employed DESeq2 analysis to compare gene expression in HFHSD and CD groups. cDC1 expression profile was similar between groups, with no significantly different expressed genes (Figure 4e).

Taken together, these data indicate that HFHSD does not lead to significantly altered cDC1 genetic profiles when compared to CD conditions.

### 2.5. Oral Tolerance is Increased in Obesity

cDC1s are essential to drive both CD8^+^ and CD4^+^ T cell activation [4]. Therefore, we tested if cDC1‐T cell interactions could be altered in obesity. We identified intestinal CD4^+^ T cell sub‐populations within the CD4^+^ T cell cluster and used CellChat to conduct a systems‐level analysis of cell–cell interactions between CD4^+^ T cell sub‐populations and cDC1s (Supporting Information 5: Figure S5a,b). CellChat predicted the most pronounced differential interaction received by cDC1s (upregulated) and Th1 cells (downregulated) when comparing HSHFD when CD‐fed mice. This suggests that HFHSD influences interactions between cDC1s and CD4^+^ T cells with an increase in number and strength of interactions towards cDC1s (Figure 5a,b).

Figure 5Functional impact of HFHSD shaped enteric immune landscape. (a, b) Differential cell–cell interactions predicted by CellChat between CD4^+^ T cells and cDC1 in HFHSD compared to CD‐fed mice in scRNA‐seq dataset from Wang et al. [1]. Violet colour indicates downregulated interaction; pink colour indicates upregulated interaction. (c) Experimental setup for food tolerance experiment: Male wild‐type mice were fed HFHSD or CD for 8 weeks and adoptively transferred with 1 × 10^6^ naïve CD45.1 CD4^+^ OT‐II T cells. Mice received two doses of intragastric OVA at 48 and 24 h before analysis. (d) Percentage of cells in OT‐II^+^ cells from mesenteric lymph nodes (*n* = 15). Data are pooled from three independent experiments. *n* represents biologically independent animals. Data are presented as mean ± s.e.m. Two‐tailed unpaired Student’s *t*‐test.  ^∗^ *p* < 0.05;  ^∗∗^ *p* < 0.01;  ^∗∗∗^ *p* < 0.005;  ^∗∗∗∗^ *p* < 0.001. ns, not significant.

**Figure:**
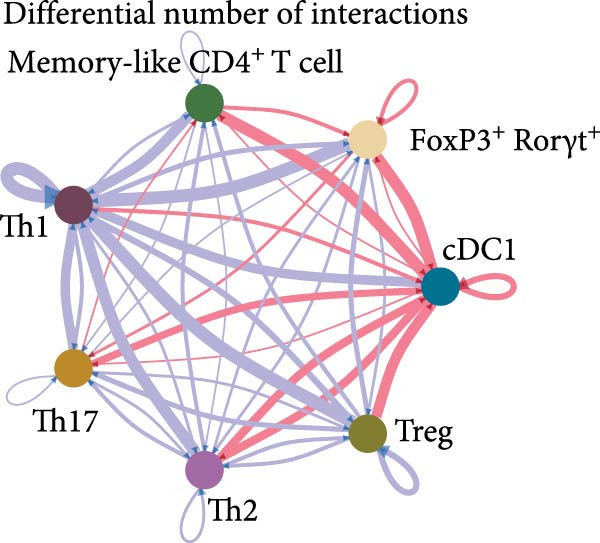
(a)

**Figure:**
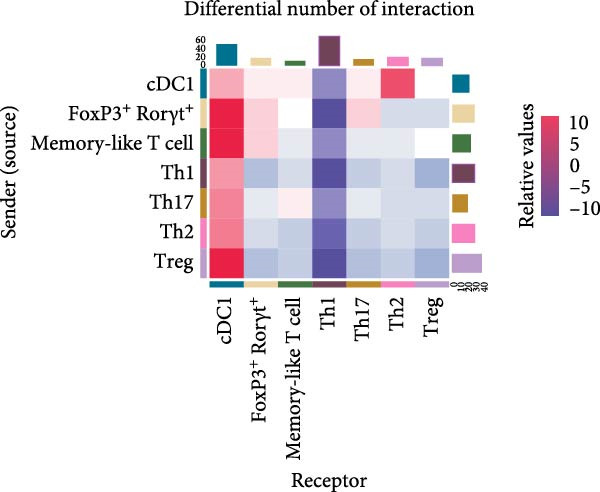
(b)

**Figure:**
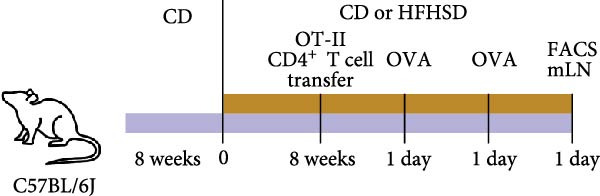
(c)

**Figure:**
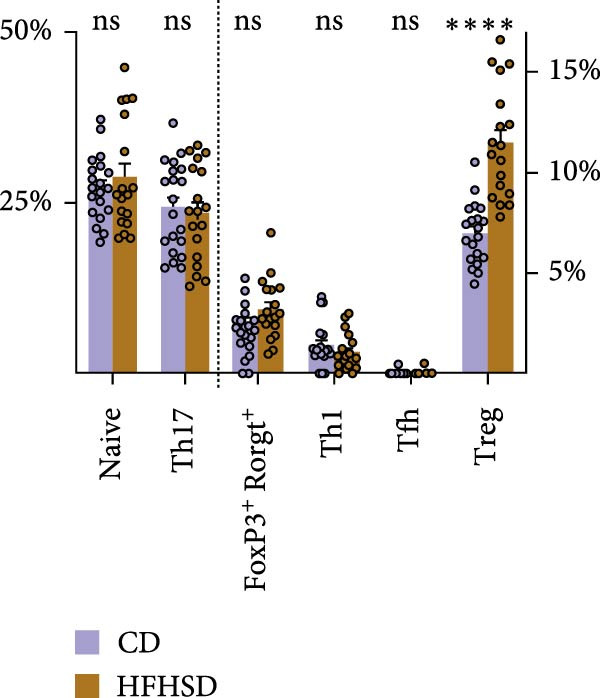
(d)

We next tested whether these predicted alterations in cDC1/CD4^+^ T cell interactions had functional consequences in vivo. Given the role of cDC1s in oral tolerance induction by promoting Tregs, we examined the impact of HFHSD‐increased enteric cDC1s on oral tolerance [9]. To this end, obese and control mice were adoptively transferred with naïve transgenic OT‐II CD4^+^ T cells that can recognise the ovalbumin (OVA) Class II peptide. Sequentially, mice were provided OVA 16–18 h after OT‐II adoptive transfer, followed by a second antigen dose 24 h after the first OVA administration. A total of 24 h after the second OVA administration and analysed 24 h after that. We found mLN Tregs to be significantly increased in obese mice, suggesting enhanced tolerogenic response to oral antigen upon HSHFD (Figure 5c,d).

In sum, our results indicate that HFHSD reshapes the enteric immune landscape. Obese mice have a higher frequency of intestinal cDC1s and interaction between these cells and CD4^+^ T cells is predicted to be affected by diet. This obesogenic immunophenotype has physiologic relevance in the context of oral tolerance, with enhanced generation of Tregs upon food‐antigen challenge.

## 3. Discussion

Our study revealed that the enteric immune system adapts to an obesogenic diet as rapidly as 4 weeks upon dietary intervention and remained stable throughout the 16‐week duration of the study. PC analysis indicated the dietary regimen to be the main driver of enteric immune alterations, and not duration of dietary intervention, as attested by clustering of immunophenotypes dependent on diet but not on duration of dietary intervention (Figure 1b). Given that body weight significantly increased between each time point (4, 8 and 16 weeks) in the HFHSD group, which is also accompanied by progressive worsening of metabolic syndrome [19], our data suggests that the enteric immune system actively establishes a new homeostatic set point rather than progressively adapting in response to high‐caloric dietary changes. Moreover, the observed increased variability under CD suggests that intestinal immunological plasticity is greater in the healthy state than in obesity as reported [20].

Regional differences of intestinal immune composition have been described previously, but a systematic comparison of all immune cell types across intestinal segments in mice has been lacking until now [21]. Our analysis revealed that all major immune cell types, except for CD4^+^ T cells, are significantly influenced by their intestinal location in steady state (Supporting Information 1: Figure S1c). We identified more similar immunophenotypes in duodenum and jejunum compared to the ileal immunophenotype, consistent with the reported peculiarities of the distal small intestine [21]. Our data indicated that immunophenotypes of specific intestinal segments remained similar to their counterparts between dietary conditions than to other segments within the same diet. We also found that T cell subtypes in the epithelium were more affected by diet compared to those in the lamina propria, possibly reflecting the unique position of the epithelium as the primary interface between the host immune system with dietary antigens and the microbiota.

The most notable finding of our screen was the significant increase in cDC1 frequency across intestinal segments in response to HFHSD, which was accompanied by a previously reported decrease in CD8^+^ T cells [1]. This interdependence is highlighted by previous studies, in which lack of T lymphocytes led to increased enteric cDC1s, whereas absence of cDC1s leads to diminished intestinal T cells [22, 23]. XCR1–XCL1 axis is essential for this cross‐talk, with CD8^+^ T cells being the main source of XCL1. XCL1 deficient mice show diminished intestinal T cells along with an accumulation of cDC1s, whereas blocking of XCL1 with antibody leads to an increase in intra‐epithelial CD8αα^+^ T cells and lamina propria CD4^+^ T cells [23].

cDC1 accumulation in the small intestine was not accompanied by changes in BM CDPs and pre‐cDCs, which contrasts to the described substantial BM remodelling in obesity, including expanded myeloid‐biased haematopoiesis and cDCpoiesis [15, 24]. Moreover, our observation of unperturbed pre‐cDC1 levels in blood argues against increased BM progenitor proliferation and/or egress that could justify elevated enteric cDC1 upon HFHSD.

The homing process of cDCs to the small intestine involves the gut‐homing markers α4β7 and CCR9, whose levels were unchanged between our dietary experimental conditions [18]. However, cDC1s also require vitamin A, the chemokine XCL1, and Flt3L to be attracted to the small intestine. While we observed fewer intestinal CD8^+^ T cells in obesity, previous publications demonstrated that these cells up‐regulate *Xcl1* expression upon HFHSD [1]. Our finding of stable total jejunal *Xcl1* mRNA levels could suggest a compensatory mechanism where reduced T cell numbers are offset by increased per‐cell *Xcl1* production. XCL1‐signalling is also critical for migration of intestinal cDC1s to the mLNs as XCL1‐ and XCR1‐deficient mice have more cDC1s in the small intestine and less cDC1s in mLNs [22]. Our data showed elevated cDC1 frequencies in both intestine and mLNs in the obese state, suggesting unaltered cDC1 migration out of the intestines. Lastly, Flt3L, a ligand that can be modulated by diet, affects DC development and local DC division [3]. We found no evidence for altered local or systemic Flt3L levels upon HFHSD along with no enhanced local cDC1 proliferation in the intestine of obese mice. The possibility that differential survival rates contribute to cDC1 accumulation remains to be explored in additional studies.

cDCs are constantly replenished from precursors arriving through the blood and migrating unidirectionally from tissues to lymph nodes, utilising chemokine signals like the CCR7‐CCL19/21 axis [25]. Reverse trafficking from lymph nodes to tissues or direct migration between peripheral organs is not documented to our knowledge, but our observation of reciprocal changes in cDC1 frequencies between liver and small intestine during obesity raise intriguing questions about potential inter‐tissue trafficking.

Recent studies have demonstrated that lipid and cholesterol exposure typically triggers homeostatic cDC maturation, resulting in a distinct tolerogenic phenotype [26]. However, HFHSD‐cDC1s in our study maintained largely normal functional characteristics, evidenced by unchanged expression of co‐stimulatory and MHC molecules, with only subtle alterations in their regulatory profile, namely downregulation of *Il10* mRNA.

Moreover, core cDC1 functions are maintained, including the expression of Treg‐inducing machinery (αvβ8 integrin and ALDH1A1). The preservation of these pathways is particularly important given their critical role in the metabolism of RA and TGF‐β activation, respectively, which are crucial in maintaining intestinal tolerance [27, 28]. The stability of the cDC1 transcriptional programme in obesity confirmed through comparison with a previously published dataset indicates remarkable functional resilience [1], suggesting possible tissue‐specific mechanisms protecting intestinal cDC1s from obesity‐induced dysfunction.

The selective increase in cDC1 frequency without major functional alterations suggests a quantitative rather than qualitative shift in intestinal immune regulation during obesity. Therefore, we reasoned this increased frequency of cDC1s in the small intestine during obesity could influence T cell responses independent of their activation state. cDC1s are specialised in cross‐presentation to CD8^+^ T cells and also support CD4^+^ T cell responses [4]. cDC frequency could affect the balance with other antigen‐presenting cells and impact T cell priming efficiency, being relevant for rare antigen‐specific T cells or the acquisition of tolerance to novel antigens [29]. Additionally, accumulated cDC1s in the intestine could activate CD4^+^ cells or interact with XCL1‐expressing CD8^+^ T cells via XCR1 and enhance CTL differentiation independently of their cross‐presentation capacity. This may influence several obesity‐associated intestinal T cell‐driven pathologies including inflammatory bowel disease (IBD), coeliac disease and gastrointestinal cancers [30, 31].

Given the reduction of CD8^+^ T cells in our settings, we focused our analysis on cDC1/CD4^+^ T cell interactions. CellChat analysis of the scRNA‐seq dataset predicted highly altered cDC1/CD4^+^ T cell interactions in obesity. In the context of food tolerance, we observed significantly enhanced tolerogenic response to oral antigens in obese mice, characterised by increased oral antigen‐specific Treg frequency. While this could be related to the impact of inflammatory‐driven changes in the mLN cDC1/cDC2 ratios on oral tolerance as recently described [9], further investigation is needed to establish a causal link between these two phenomena, and to define the contribution of RORgt‐expressing APCs to this process, which have recently emerged as key regulators of both microbiota‐ and food antigen‐induced pTreg differentiation [32–35].

Despite the parallel increases in obesity and food intolerances, there is limited knowledge about the relation between those diseases and their underlying mechanisms. While the relationship between enhanced oral tolerance observed in our study and the worldwide increase in food allergies concurrent with rising obesity rates might be seen as paradoxical, these differences might be explained by the distinct immunological outcomes in adult‐ versus early‐life obesity, prenatal and postnatal dietary habits of the mother and microbial factors, significantly influencing the development of oral tolerance [36]. Future experiments are needed to understand the correlation between increased obesity and food intolerances, as well as the impact of enhanced tolerance that we observed on immune responses to pathogens and the risk of obesity‐associated cancers.

Our spatio‐temporal analysis of the enteric immune landscape in obese animals revealed that enteric cDC1s accumulated upon HFHSD independently of changes of BM precursors, and gut homing capacity, and did not show altered activation status nor cytokines production. These features were accompanied by enhanced oral tolerance to food antigens demonstrated by significant increase of antigen specific Tregs. This newly identified mechanism to cope with chronic metabolic stress while maintaining tissue homeostasis will open new avenues for understanding how the immune system adapts to the worldwide rising prevalence of obesity.

## 4. Materials and Methods

### 4.1. Experimental Animals

All mouse lines were bred on a full C57BL/6J background. Mice were housed and bred at Champalimaud Centre for the Unknown (CCU) under specific pathogen‐free conditions. Mice were maintained on 12 h light–dark cycles, with access to food and water ad libitum, if not stated otherwise. All used mice were male and age matched. All animal experiments were approved by national and local institutional review boards (IRBs), Direção Geral de Veterinária and CCU ethical committees. C57BL/6J mice were purchased from Charles River and maintained in our facilities. OT‐II TCR‐transgenic mice were originally purchased from Jackson Laboratories, crossed with C57BL/6 Ly5.1 and with Rag1^−/−^ and maintained in our facilities [37, 38]. Further details are provided in Table 1.

**Table 1:** Experimental animals, reagents and materials.

Resource	Source	Identifier
Experimental animals		
Mouse: C57BL6/J (CD45.2)	Charles River	632C57BL/6J
Mouse: C57BL6/J (CD45.1)	Jackson Laboratories	2014
Mouse: C57BL6/J Rag1^−/−^	Jackson Laboratories	2216
Mouse: OTII	Jackson Laboratories	4194
Dietary intervention		
CD	SDS	801030
HFHSD	Ssniff GMbH	E15742‐347
Glucose	Fisher Scientific	10539380
Fructose	Sigma–Aldrich	F3510
Glucose tolerance tests		
Glucose	Fisher Scientific	10539380
PBS	Corning	46‐013‐CM
Glucometer	Roche	7819382037
Accu‐Chek Aviva Test Stripes	Roche	6453970023
Cell isolation and purification		
Collagenase D	Roche	11088882001
Collagenase II	Gibco	LTI 17101‐015
Collagenase VI	Worthington	LS004188
DMEM	StemCell	36254
DNase I	Roche	10104159001
DTT	Sigma–Aldrich	43816‐0010
EDTA	Corning	46‐034‐CI
FBS	Corning	35‐079‐CV
HBSS	Corning	21‐023‐CV
Heparine	Braun	P8721
HEPES	Corning	25‐060‐CI
L‐glutamine	Corning	10‐040‐CVR
Liberase TL	Roche	5401020001
Liberase TM	Roche	5401127001
PBS	Corning	46‐013‐CM
Penicillin/streptomycin	Corning	30‐002‐CI
Percoll	Cytiva	GE17‐0891‐01
RBC lysis buffer	eBioscience	00‐4300‐54
RPMI	Corning	10‐040‐CVR
Sodium pyruvate	Corning	25‐000‐CIR
β‐Mercaptoethanol	Gibco	31350‐010
Cell stimulation and staining		
Brefeldin A	BD Horizon	420601
Anti‐CD16/32 (2.4G2)	Invitrogen	14‐0161‐86
Brilliant Stain Buffer	BD Horizon	566385
LIVE/DEAD Fixable Aqua Dead Cell Stain Kit	eBioscience	00‐8222‐49
IC fixation/permeabilization kit	Invitrogen	00‐8222‐49
Antibodies for cell staining		
Anti‐mouse B220 FITC	BioLegend	103206
Anti‐mouse c‐Kit APC Cy‐7	eBioscience	17‐1171‐82
Anti‐mouse Ccr9 Pe Cy‐7	BioLegend	128712
Anti‐mouse c‐Kit APC	eBioscience	17‐1171‐82
Anti‐mouse CD103 BV605	BioLegend	121433
Anti‐mouse CD115 BV605	BioLegend	135517
Anti‐mouse CD11b BV785	BioLegend	101243
Anti‐mouse CD11b FITC	BioLegend	101206
Anti‐mouse CD11b BV785	BioLegend	101243
Anti‐mouse CD11c PE	BioLegend	117308
Anti‐mouse CD11c BV650	BioLegend	117339
Anti‐mouse CD11c FITC	BioLegend	117306
Anti‐mouse CD127 BV421	BioLegend	135024
Anti‐mouse CD150 Pe Cy‐7	eBioscience	25‐1502‐82
Anti‐mouse CD172 AF700	BioLegend	144021
Anti‐mouse CD19 FITC	BioLegend	115506
Anti‐mouse CD19 BV605	BioLegend	115540
Anti‐mouse CD25 PerCP Cy‐5.5	Invitrogen	45‐0251‐82
Anti‐mouse CD25 BV711	BioLegend	102049
Anti‐mouse CD27 PE	Invitrogen	12‐0271‐82
Anti‐mouse CD34 BV421	BioLegend	119321
Anti‐mouse CD3ε FITC	BioLegend	100306
Anti‐mouse CD3ε Biotin	BioLegend	100304
Anti‐mouse CD4 BV785	BioLegend	100551
Anti‐mouse CD4 BV421	BioLegend	100437
Anti‐mouse CD4 BV650	BioLegend	100555
Anti‐mouse CD40 PerCP Cy‐5.5	BioLegend	124623
Anti‐mouse CD43 Biotin	eBioscience	13‐0341‐82
Anti‐mouse CD44 FITC	BioLegend	103006
Anti‐mouse CD45.1 BV650	BioLegend	110735
Anti‐mouse CD45.2 APC	BioLegend	109814
Anti‐mouse CD45.2 BV711	BD Horizon	563685
Anti‐mouse CD45.2 Pe Cy‐7	BioLegend	109830
Anti‐mouse CD45.2 AF700	Invitrogen	56‐0454‐82
Anti‐mouse CD45.2 APC Cy‐7	BioLegend	109824
Anti‐mouse CD48 AF647	BioLegend	103416
Anti‐mouse CD49b PE‐ef610	Invitrogen	61‐5971‐82
Anti‐mouse CD5 FITC	Invitrogen	11‐0051‐82
Anti‐mouse CD64 BV711	BioLegend	139311
Anti‐mouse CD64 FITC	BioLegend	139316
Anti‐mouse CD80 FITC	BioLegend	104706
Anti‐mouse CD86 PE Cy‐5	BioLegend	105016
Anti‐mouse CD8α Bv711	BioLegend	100747
Anti‐mouse CD8α FITC	BioLegend	100706
Anti‐mouse CD8α APC	BioLegend	100712
Anti‐mouse CD8β PE	Invitrogen	12‐0081‐83
Anti‐mouse CXCR5 APC/Fire750	BioLegend	126539
Anti‐mouse Flt3 Pe	BioLegend	135306
Anti‐mouse Flt3 Pe Cy‐5	BioLegend	135312
Anti‐mouse FoxP3 APC	Invitrogen	17‐5773‐80
Anti‐mouse GM‐CSF PerCP Cy‐5.5	BioLegend	505410
Anti‐mouse Gr1 FITC	BioLegend	108406
Anti‐mouse Il‐6 APC	BioLegend	505410
Anti‐mouse Il‐10 Pe Cy‐7	BioLegend	505025
Anti‐mouse Il‐12 PE	BD Bioscience	554480
Anti‐mouse KLRG1 BV421	BioLegend	138414
Anti‐mouse Ly6C PE	Abcam	ab25572
Anti‐mouse Ly6D FITC	BioLegend	138606
Anti‐mouse Ly6G FITC	BioLegend	127606
Anti‐mouse Ly6G PE	BioLegend	127607
Anti‐mouse MHCII APC Cy‐7	BioLegend	107628
Anti‐mouse MHCII FITC	BD Bioscience	562009
Anti‐mouse MHCII PerCP	BioLegend	107624
Anti‐mouse MHCI eF450	Invitrogen	48‐5958‐82
Anti‐mouse NK1.1 FITC	Invitrogen	11‐5941‐85
Anti‐mouse NK1.1 Pe Cy‐7	Invitrogen	25‐5941‐82
Anti‐mouse Nkp46 PerCP ef710	Invitrogen	46‐3351‐82
Anti‐mouse Rorγt PE	Invitrogen	12‐6988‐82
Anti‐mouse Sca‐1 BV785	BioLegend	108139
Anti‐mouse Siglec‐H PB	BioLegend	129610
Anti‐mouse SiglecF FITC	BioLegend	155504
Anti‐mouse T‐bet PerCP Cy‐5.5	Invitrogen	45‐5825‐82
Anti‐mouse TCRVα2 ef450	Invitrogen	48‐5812‐82
Anti‐mouse TCRβ PE Cy‐5	BioLegend	109209
Anti‐mouse TCRβ FITC	BioLegend	109206
Anti‐mouse TCRγδ PE Cy‐7	Invitrogen	25‐5711‐82
Anti‐mouse TCRγδ FITC	BioLegend	118106
Anti‐mouse Ter119 FITC	BioLegend	116206
Anti‐mouse Thy1.2 Bv605	BioLegend	140318
Anti‐mouse TNFα BV421	BioLegend	506328
Anti‐mouse XCR1 af647	BioLegend	148213
Anti‐mouse α4β7 APC	BioLegend	120607
Anti‐biotin Streptavidin BV421	BD Horizon	563259
Anti‐biotin Streptavidin BV605	BioLegend	405229
RNA extraction and RT‐qPCR		
RNeasy Plus Mini Kit	Qiagen	50974136
RNase‐Free DNase Set	Qiagen	50979254
Ethanol	VWR Chemicals	VWRC20821.330
PreAmp Master Mix	Applied Biosystems	LTAB 4488593
TaqMan Gene Expression Master Mix	Applied Biosystems	LTAB 4370074
Aldh1a1	Thermo Scientific Fisher	Mm00657317_m1
Gapdh	Thermo Scientific Fisher	Mm99999915_g1
Hprt	Thermo Scientific Fisher	Mm03024075_m1
Itgb8	Thermo Scientific Fisher	Mm00623991_m1
Il10	Thermo Scientific Fisher	Mm00439614_m1
Il12a	Thermo Scientific Fisher	Mm00434169_m1
Il15	Thermo Scientific Fisher	Mm00434210_m1
Tnfa	Thermo Scientific Fisher	Mm00443260_g1
Tgfb1	Thermo Scientific Fisher	Mm01178820_m1
Elisa		
2‐Mercaptoethanol	Gibco	31350‐010
BCA Protein Assay Kits	Pierce	23225
DuoSet ELISA Ancillary Reagent Kit 2	Biotechne	DY008B
EDTA	Corning	46‐034‐Cl
EGTA	Sigma–Aldrich	E3889
Mouse FLT3L DuoSet ELISA	Biotechne	DY427
NaF	Sigma–Aldrich	201154
PMSF	Sigma–Aldrich	P7626
Protease inhibitor cocktail	Roche	11836153001
Pyrophosphate	Sigma–Aldrich	221368
Sodium glycerophosphate	Sigma–Aldrich	61668
Sodium orthovanadate	Sigma–Aldrich	567540
Sucrose	Sigma–Aldrich	S9378
Tris‐HCL	Fisher Scientific	J/4315/17
Triton X‐100	Fisher Scientific	BP151‐500
Food tolerance experiment		
Anti‐mouse B220 Biotin	eBioscience	13‐0452‐82
Anti‐mouse CD11b Biotin	Invitrogen	13‐0112‐85
Anti‐mouse CD11c Biotin	Invitrogen	13‐0114‐85
Anti‐mouse CD19 Biotin	BioLegend	115504
Anti‐mouse CD25 BV711	BioLegend	102049
Anti‐mouse CD45.1 Biotin	eBioscience	13‐0453‐82
Anti‐mouse CD45.1 BV650	BioLegend	110735
Anti‐mouse CD45.2 PE Cy‐7	BioLegend	109830
Anti‐mouse CD8α Biotin	Invitrogen	13‐0081‐85
Anti‐mouse Gr‐1 Biotin	Invitrogen	13‐5931‐85
Anti‐mouse NK1.1 Biotin	Invitrogen	13‐5941‐85
Anti‐mouse TCRVα2 ef450	Invitrogen	48‐5812‐82
Anti‐mouse TCRVβ5 PE	BioLegend	139501
Anti‐mouse Ter‐119 Biotin	eBioscience	13‐5921‐85
MACS beads	Milteny Biotec	130‐048‐102
LS columns	Milteny Biotec	130‐042‐401
QuadroMAC Separator	Milteny Biotec	130‐091‐051
OVA (Grade V)	Sigma–Aldrich	A5503
Isoflurane	Zoetis	571329,8

### 4.2. Dietary Intervention

Mice were raised on a standard chow diet until they reached 8 weeks of age. Adult mice were randomly assigned to one of two dietary regimens: a HFHSD or a continuation of the normal chow as a CD as previously described. In short, HFHSD included 60% of calories from fat (lard), corresponding to the D12492 formulation and drinking water was supplemented with 23.1 g/L fructose (55% *w*/*w*) and 18.9 g/L glucose (45% *w*/*w*), resulting in a total sugar concentration of 42 g/L. The control group continued the standard CD, which contained 12% calories from fat‐origin, and normal, non‐supplemented drinking water. Mice had access to food and drinking ad libitum. More information is provided in Table 1.

### 4.3. Glucose Tolerance Test

Glucose tolerance in mice was evaluated 1 week prior to experiments. Following an overnight fast, mice were administered an intra‐peritoneal injection of glucose (2 mg/kg body weight) dissolved in PBS. Blood glucose levels were measured using an ACCU‐CHEK Aviva glucometer immediately before glucose administration and at 15‐min intervals for 2.5 h post‐injection. Additional information is provided in Table 1.

### 4.4. Cell Isolation and Purification

#### 4.4.1. Small Intestine and Colon

For the examination of the small intestinal immune system in obesity and steady‐state conditions, mice were fasted overnight (8 h) prior to euthanasia. Small intestines were opened longitudinally and divided into three sections: duodenum (proximal 25% of the intestine), jejunum (medial 50% of the intestine) and ileum (distal 25% of the intestine). Each section was cut into 1 cm long pieces and processed as previously described [39]. In brief, tissues were exposed to RPMI supplemented with 5% FBS, 1% HEPES, 1% sodium pyruvate, 1% streptomycin and penicillin and 0.1% β‐mercapto‐ethanol (cRPMI) containing 5 mM DTT in an orbital shaker at 700 × g for 20 min. Supernatant was removed, and cRPMI 5 mM EDTA was added and agitated at 700 × g for 20 min at 37°C twice. For epithelial cell isolation, supernatants were collected. For lamina propria cells isolation, the intestinal pieces were washed minced and digested with DNase I (20 U/mL) and Collagenase D (5 mg/mL) or Liberase TM (25 μg/mL) for cDC isolation for 30 min at 200 rpm 37°C. The reaction was halted by adding 10 mL of ice‐cold cRPMI. The resulting cell suspension was passed three times through an 18G needle. For lymphocyte analysis, the digests were purified by centrifugation for 30 min at 1200 × g in a 40/80 Percoll gradient.

Colon was cut longitudinally and into 1 cm pieces and incubated with PBS supplemented with 5% FBS, 2 mM EDTA and 1 mM DTT at 37°C 700 × g for 15 min. For enzymatic digestion, the tissue was incubated in 10 mL of HBSS supplemented with 1% FBS, Collagenase IV (0.5 mg/mL) and DNase I (20 U/mL) for 45 min at 700 × g at 37°C. The reaction was stopped by adding cRPMI.

#### 4.4.2. Liver, Pancreas and Fat

Following perfusion, the liver was digested wit Collagenase IV (0.5 mg/mL) and DNase I (20 U/mL) at room temperature for 20 min and treated as previously described [39]. Following ductal perfusion, pancreas was digested with Collagenase D (5 mg/mL) and DNase I (20 U/mL) for 30 min with constant agitation at 1100 rpm at 37°C. Fat tissue was treated as previously described [16]. In brief, tissue was digested by Collagenase II (3 mg/mL) at 37°C for 20 min.

#### 4.4.3. Spleen and Lymph Nodes

Spleen was digested with Liberase TL (5 mg/mL) and DNase I (20 U/mL) in two 15 min incubations at 37°C. Lymph nodes were treated with Collagenase D (400 U/mL) for 25 min at 37°C and mechanically disrupted by passing it three times through an 18G syringe needle.

#### 4.4.4. BM and Blood

Bones were cut open and placed into an Eppendorf tube. Bones were centrifuged for 30 s at 10,000 × g at 4°C to extract BM. Blood was collected by cardiac puncture with heparin pre‐coated syringes. Blood was centrifuged at 350 × g for 10 min at room temperature. Erythrocytes were lysed using RBC lysis buffer.

### 4.5. Flow Cytometry

For cytokine analysis of cDCs, mice were injected i.p. with Brefeldin A (125 μg/mouse) in 100 μL PBS 12 h prior to analysis.

Permeabilization and fixation of cells for intracellular staining were performed using an IC fixation/permeabilization kit for cytokine and transcription factor analysis. For blocking nonspecific antibody binding, cells were pre‐incubated with anti‐CD16/32 (1:200) in PBS for 10 min at 4°C. Afterwards, samples were stained for 20 min at 4°C with an antibody cocktail and LIVE/DEAD Fixable Aqua Dead Cell Stain Kit in PBS. When necessary, Brilliant Stain Buffer was added to the staining medium. For intra‐cellular staining, cells were incubated overnight with intra‐cellular antibodies and acquired on the next day.

Cell populations were gated on live cells and defined as ILC2: CD45+Lin−Thy1.2+ Sca‐1+KLRG1+; ILC3: CD45+Lin−Thy1.2highRORγt+ (lineage comprised CD3ε, CD8α, TCRβ, TCRγδ, CD19, GR1, CD11c, CD11b and TER119); NK: CD45+Lin− NK1.1+NKp46+CD27+CD49b+CD127−; ILC1: CD45+Lin−NK1.1+NKp46+CD27+ CD49b−CD127+ (lineage without CD11b and NK1.1); B cell: CD45+TCR−CD19+; CD4+ TCRβ+ T cell: CD45+TCRβ+CD4+; CD4+ TCRγδ+ T cell: CD45+TCRγδ+CD4+; CD8α+TCRβ+ T cell: CD45+TCRβ+CD8α+; CD8α+TCRγδ+ T cell: CD45+TCRγδ+ CD8α+; cDC: CD45+CD64−MHCII+CD11c+; cDC1: CD45+CD64−MHCII+CD11c+ CD11b−CD8α+ or CD45+CD64−MHCII+CD11c+Xcr1+Sirplow cDC2: CD45+CD64− MHCII+CD11c+CD11b+CD8α‐ or CD45+CD64−MHCII+CD11c+Xcr1‐Sirphigh; neutrophil: CD45+CD3ε−CD64−Ly6G+CD11b−; macrophage and transitional monocyte: CD45+CD11b+CD11clowMHCII+; monocyte: CD45+CD11b+CD11clow MHCII−; CD8αα+TCRβ+ T cell: CD45+CD8α+CD8β−TCRγδ−TCRβ+CD4−; CD8αα+TCRγδ+ T cell: CD45+CD8α+CD8β−TCRγδ+TCRβ−CD4−; dpIEL: CD45+ CD8α+CD8β−TCRγδ−TCRβ+CD4+; CD8αβ+ T cell: CD45+CD8α+CD8β+; Treg: CD45+MHCII−CD19−CD8α−CD8β−CD4+TCRβ+CD25+FoxP3+. Lineage for DC progenitors included: CD3ε, CD19, NK1.1, B220, Ly6D, Ly6G, SiglecF and Ter119 and cells were defined as CDP: lin‐CD115+ CD135+CD117low; pre‐cDC: CD45+CD11c+Lin‐MHCII‐CD11blow/intSirpalowFlt3+ CD43+; pre‐cDC1: CD45+CD11c+lin‐MHCII‐CD11blow/intSirpalowFlt3+CD43+ SiglecH‐Ly6C‐; pre‐cDC2: CD45+CD11c+lin‐MHCII‐CD11blow/intSirpalowFlt3+ CD43+SiglecH‐Ly6C+; uncommitted pre‐cDC: CD45+ CD11c+lin‐MHCII‐CD11blow/intSirpalowFlt3+CD43+SiglecH+Ly6C‐. OT‐II T cells were defined as CD45.1+CD4+ TCRVα2+, encompassing naïve (CD44‐), Tregs (FoxP3+), Tfh (CXCR5+), Th1 (Tbet+), Th17 (Rorγt+) and FoxP3+Rorγt+ cells.

Stained cells were acquired in FACS buffer with an LSRFortessa X‐20 Flow cytometer (BD Biosciences), and cell sorting was performed using a FACSFusion (BD Biosciences). Sorted cell populations were >95% pure. Data were analysed using FlowJo 10.10.0 software (Tree Star). All cell populations were gated in live cells (Aqua‐negative cells). Information regarding the antibodies is provided in Table 1.

### 4.6. Quantitative PCR With Reverse Transcription (RT‐PCR)

Total RNA extraction in sorted cells was performed using the RNeasy mini kit, following the manufacturer’s protocol. The concentration and purity of the isolated RNA were determined after the extraction using a Nanodrop Spectrophotometer (Nanodrop Technologies).

Quantitative real‐time RT‐PCR was performed in a QuantStudio 5 real‐time PCR system (Applied Biosystems) with *Hprt* and *Gapdh* as housekeeping genes. To retro‐transcribe RNA, a High‐Capacity RNA‐to‐cDNA Kit was used. Afterwards, pre‐amplification PCR was performed using TaqMan PreAmp Master Mix. A TaqMan Gene Expression Master Mix was used for the real‐time PCR. The following TaqMan Gene Expression Assays were used: *Tgfb1*, *Il10*, *Il12a*, *Il15*, *Itgb8*, *Aldh1a1*, *Tnfa*, *Hprt* and *Gapdh*. Analysis was performed with the comparative CT method (2*Δ*CT), and comparison or fold change between samples was assessed with the comparative *Δ*CT method (2*ΔΔ*CT) [40]. Further Taqman probes information in Table 1.

### 4.7. Flt3l Elisa

Tissues were weighed and collected in 1 mL Triton X‐100 lysis buffer (50 mM Tris‐HCl pH 7.5, 1 mM EGTA, 1 mM EDTA pH 8.0, 50 mM NaF, 1 mM sodium glycerophosphate, 5 mM pyrophosphate, 0.27 M sucrose 0.5% Triton X‐100, 0.1 mM PMSF, 0.1% 2‐mercaptoethanol, 1 mM sodium orthovanadate and protease inhibitor cocktail [Roche]). One stainless steel bead was added per sample, and samples were disrupted with a TissueLyser (Qiagen) at 30 Hz for 10 min. Samples were centrifuged at 16,000 × g for 15 min at 4°C to pellet cellular debris and supernatant was collected. To obtain serum, blood was collected by cardiac puncture and with syringes that were pre‐coated with heparin. Samples were centrifuged at room temperature at 200 × g for 10 min and serum was collected.

Elisa was conducted according to the manufacturer’s protocol. Optical density was measured with a SPARK plate reader set to 450 nm. For correction of optical imperfections in the plate, readings at 540 nm were subtracted from the readings at 450 nm. Protein was normalised to total protein measured using a BCA protein assay kit.

### 4.8. Food Tolerance OVA Model

For antigen challenge, OVA was administered intragastrically (50 mg in 200 μL PBS 1x) using plastic gavage needles. Mice were challenged twice, once 16–18 h after adoptive OT‐II cell transfer and the second time 24 h after the first OVA administration. Animals were analysed 24 h after the second OVA administration.

For adoptive T cell transfer, naïve CD4^+^ OT‐II T cells were negatively selected using biotinylated antibodies against CD8α, CD25, CD11c, CD11b, TER‐119, NK1.1, and B220 and Streptavidin MACS beads. The purity of transgenic was verified by flow cytometry (typically >80% of cells were CD45.1^+^Vα2^+^Vβ5^+^CD25). 1x10^6^ naïve CD4^+^ OT‐II cells were transferred by retro‐orbital injection.

### 4.9. Dimensionality Reduction

The PCA was done following the Eigen‐decomposition method. For that, first, the mean cell count ratio over all animals that underwent the same staining was computed. This mean would contain a value per cell type and per week, segment and diet. Since the data was already a ratio, no further normalisation was done besides centring the data. Centring was done by subtracting the overall mean value per cell type across all weeks, all segments, and all diets from the previously obtained means, this gave us the matrix *X*, which was used for the PCA.

The PCA was computed using the sklearn python package, which computes the variance (*V*) of the centred data and does the eigenvalue decomposition of such variance.

V=WDWT.

PCA separates the data into two matrices: *D* and *W*. *D* is a diagonal matrix in which each of the diagonal entries is the variance explained by each of the principal components. Each column of the *W* matrix () corresponds to one component, in which each entry represents the weight of each of the cell types.

The projections of the principal component were then computed, projecting these weights back on the data (*X*).

PCi=Xwi.

Each of these projections is a one‐dimensional vector with a value for each of the weeks, the segments, and the diets. In the analysis, the first three projections were used to visualise a summary of the data. Colouring for diet type and week or diet and segment allowed us to visually inspect how separable the data is. The bigger the distance between points of different colours, the larger the separability.

### 4.10. Multilinear Regression

The multilinear regression was done using the sklearn python package. For every computed regression, the dependent variable was the ratio of the number of cells for each staining and the predictor of the type of diet (1 for HFHSD and 0 for CD).

The data was separated into test (20% of the data) and train (80% of the data) sets. The train set was used to calculate the value of the regressors (the weight of each of the cell types). The higher the value of the regressor for a particular cell type, the more its value increased in the HFSHSD. At the same time, the more negative the value, the more it decreased its value in the HFHSD in comparison to the CD. Since the value of many cell types can be correlated, ridge regression was calculated. This type of multi‐linear regression punishes high regressor values; therefore, only leaving regressors that have the strongest relationship with the predictor. The value of the penalisation was 0.01.

Once the ridge regression was calculated on the train data set, the performance of this regression on the test set was calculated. This data was not seen by the model. Therefore, it provides insight into how good the obtained regressors are at predicting the diet type. Thus, the test data was used to calculate the accuracy of the model. This accuracy was calculated by the number of times the model inferred the diet correctly divided by the total number of data points. The whole procedure was repeated 100 times, and the average values of the accuracy and the regressors were shown in the figures.

### 4.11. Statistical Analysis

The statistical analysis for the screen of the immune landscape in HFHSD and CD was conducted using ANOVA and post hoc statistics. To obtain the statistical significance between the two diets and the weeks, a between‐subject ANOVA was employed, as every animal could only be a data point for a given week and diet. In the case of the segment of the intestine, a mixed ANOVA was employed, as the same animal provided one value per segment, making the intestine segment a within‐subject comparison and only one treatment, a between‐subject comparison.

In both cases, to correct for multiple comparisons, a Bonferroni correction was employed. Additionally, once the ANOVA was significant for either the diet, the week or the treatment, a post hoc analysis was performed. This analysis showed the particular combination that was significantly different.

Results in graphs are shown as mean ± s.e.m. Statistical analysis for all other experiments was conducted with GraphPad Prism software (GraphPad Software, La Jolla, Calif). Student’s *t*‐test was performed on homoscedastic populations. The Mann–Whitney test was used when data were not normally distributed. Repeated‐measures ANOVAs were performed on body weights and glucose tolerance test measurements with Sidak correction for multiple comparisons. Results were considered statistically significant at *p*  < 0.05.

### 4.12. Analysis of scRNA‐seq Dataset

Data was made publicly available by Wang et al. [1]. The output files of Cellranger were downloaded from GSE221006 (specifically GSM6837106 and GSM6837107 were used). Files were analysed in R using Seurat (v.5) following the guidelines. Data was filtered, normalised, integrated and clustered according to Wang et al. [1]. Cell types and subtypes were annotated by analysing DEGs, which were compared against established cell type definitions from the Wang et al. [1] study. DEG validation was performed using DESeq2 analysis. Sample composition analysis involved calculating the proportion of each cell type by determining cell counts per type and then converting these counts to percentages of the total cell population. CellChat version 2.1.0 was employed to analyse cellular communication patterns. The analysis merged cDC1 and CD4^+^ T cell subtypes and conducted separate analyses for intestinal cells from both CD and HFHSD conditions. Subsequently, the differential interaction between conditions was evaluated. The results visualised inter‐cellular communication patterns through heatmaps and circular interaction diagrams, highlighting both the frequency and intensity of cell–cell interactions.

## Ethics Statement

All animal experiments were approved by national and local institutional review boards (IRBs), Direção Geral de Veterinária and CCU ethical committees.

## Conflicts of Interest

The authors declare no conflicts of interest.

## Author Contributions

K. Fischer, M. Martínez‐López, O. Horno and H. Veiga‐Fernandes designed and performed the research and/or provided advice and technical expertise. K. Fischer and O. Horno analysed the data. K. Fischer and M. Martínez‐López wrote the manuscript.

## Funding

This study was funded by the Fundação para a Ciência e a Tecnologia (Grant 148058/2019), the “la Caixa” Foundation (Grant 116923), the European Research Council (Grant 647274), and the Paul G. Allen Frontiers Group.

## Supporting Information

Additional supporting information can be found online in the Supporting Information section.

## Supporting information


**Supporting Information 1** Figure S1: HFHSD leads to obesity and regional comparison of immune cell types in steady‐state. (a) Body weight of mice (*n* = 15). (b) Intra‐peritoneal glucose tolerance test (IGTT) at indicated time points (*n* = 15). (c) Lamina propria and intra‐epithelial immune cells of male wild‐type mice at 8 weeks of age as ratios of CD45^+^ cells (*n* = 15). Data are pooled from three independent experiments. *n* represents biologically independent animals. Data are presented as mean ± s.e.m. Two‐sided two‐way repeated measures ANOVA corrected for multiple comparisons (a, b). Two‐way repeated measures ANOVA for tests within subjects (c).  ^∗^ *p* < 0.05;  ^∗∗^ *p* < 0.01;  ^∗∗∗^ *p* < 0.005;  ^∗∗∗∗^ *p* < 0.001; ns, not significant.

**Supporting Information 2** Figure S2: Effect of diet, location and time on immunophenotype. (a) Ratios of selected lamina propria and intraepithelial immune cell types in CD45^+^ cells at indicated time points and intestinal segments in HFHSD or CD conditions (*n* = 15). Data are pooled from three independent experiments. *n* represents biologically independent animals. Data are presented as mean ± s.e.m (a).

**Supporting Information 3** Figure S3: Ontogeny and distribution of cDC1 in the obese and steady‐state host. Mice were fed for 8 weeks with HFHSD or CD. (a) Representative gating strategy for identification of cDC precursors by flow cytometry. (b) Total numbers of cDC precursors in bone marrow and (c) as percentage in pre‐cDCs measured by flow cytometry (*n* = 10). (d) Total numbers of pre‐cDCs and pre‐cDC1 and (e) as percentage of pre‐cDCs in the blood. (f) MFI of Ki67 of cells in the enteric lamina propria (*n* = 5). (g) cDC1 as percentage in CD45^+^ cells in metabolically relevant organs and (h) mesenteric lymph nodes (*n* = 12). (i) Representative histograms showing expression of α4β7 and CCR9 in pre‐DC1 α4β7^+^ and CCR9^+^, respectively. Flow cytometry analysis (a–i). Data are pooled from three independent experiments. *n* represents biologically independent animals. Data are presented as mean ± s.e.m. Two‐tailed unpaired Student’s *t*‐test (a–g).  ^∗^ *p* < 0.05;  ^∗∗^ *p* < 0.01;  ^∗∗∗^ *p* < 0.005;  ^∗∗∗∗^ *p* < 0.001. LP, lamina propria; ns, not significant.

**Supporting Information 4** Figure S4: Comparison of the scRNA‐seq dataset from Wang et al. [1] and our flow cytometry dataset. (a) Percentage of calories obtained from carbohydrates, fat and proteins in different diets. (b) Side‐by‐side presentation of lamina propria immune cell populations as percentages in CD45^+^ cells from mice that were fed 8 weeks HFHSD or CD. (c) Fold change of lamina propria immune cell populations from CD to HFHSD. (d) UMAP presentation of cDC subsets in scRNA‐seq dataset from Wang et al. [1]. (e) Marker gene expression used for identification of cDC1 and cDC2 in scRNA‐seq dataset. Pearson correlation (c). DESeq2 analysis (e). CCU, Champalimaud Centre for the Unknown.

**Supporting Information 5** Figure S5: CD4^+^ T cell subcluster. (a) UMAP presentation of CD4^+^ T cell cluster in scRNA‐seq dataset from Wang et al. [1]. (b) Dot plot showing selected top DEG used for identification of CD4^+^ T cell populations. DESeq2 analysis (b).

**Supporting Information 6** Raw dataset used to generate Figure 2 (Diet & Duration). Pairwise comparison of effect of diet and duration of diet on ratio of cell types in CD45^+^ cells. Two‐way repeated measures ANOVA. Rows 0–2 show pairwise comparison of duration, while not considering the treatment. Row 3 shows effect of treatment independent of duration. Rows 4–6 shows the effect of the treatment at each time point. Bonferroni correction. (Diet & Segment) Pairwise comparison of effect of diet and intestinal segment on ratios of cell types in CD45^+^ cells. Mixed‐repeated measures Anova within and between subjects. Rows 0–2 show pairwise comparison of intestinal segments while not considering the treatment. Row 3 shows effect of treatment independent of intestinal segment. Rows 4–6 show the effect of the treatment in each intestinal segment.

## Data Availability

The data supporting the findings of this study are available from the corresponding author upon reasonable request.
