# Supplementary figures and images for: High Fat and Sugar Diet Increases Enteric cDC1 and Oral Antigen‐Specific Tregs
Source: J Immunol Res. 2025 Oct 28;2025:6600076. doi: 10.1155/jimr/6600076 (PMC12564431; doi:10.1155/jimr/6600076)

**b**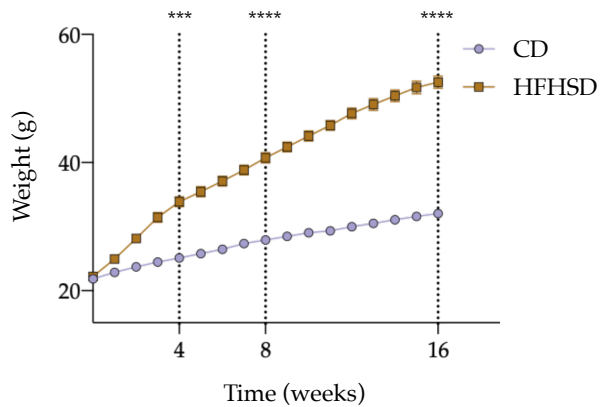**b**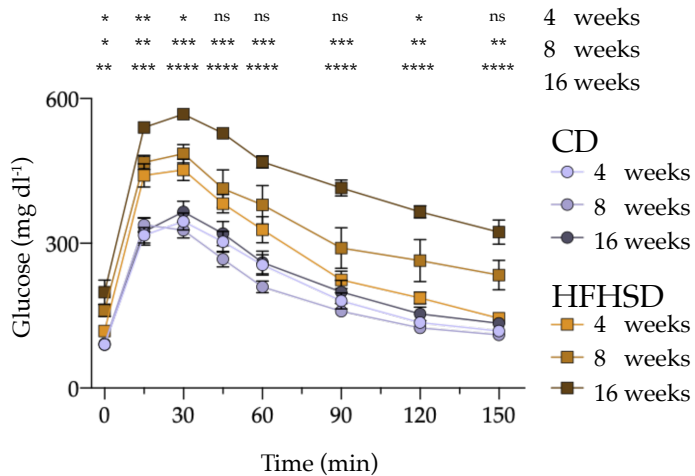

**C**

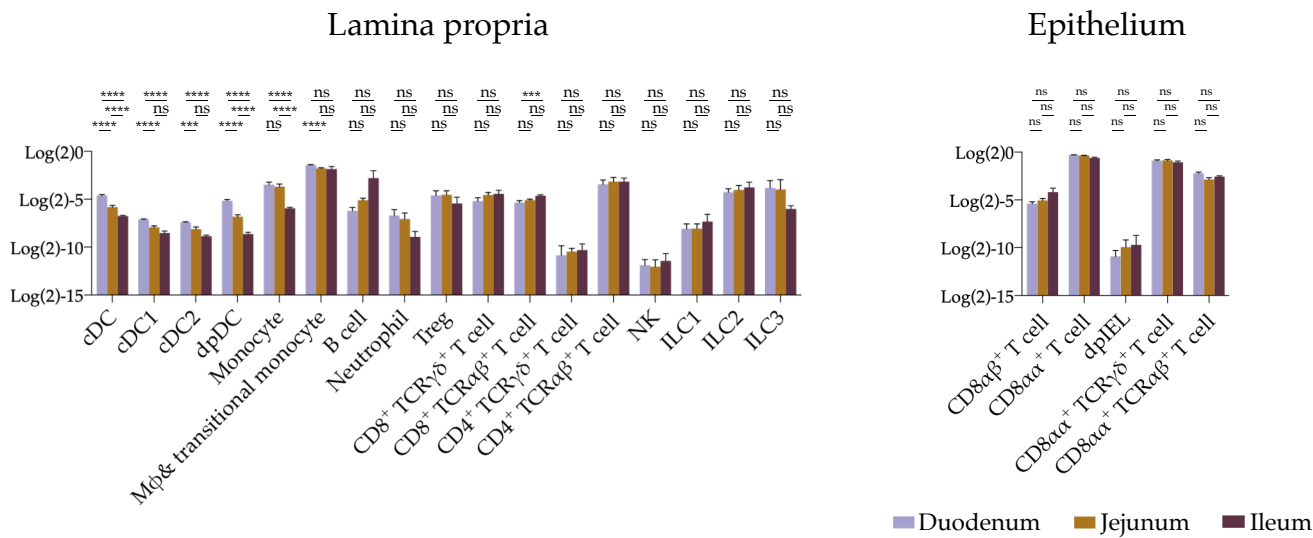

### Figure Supplementary 1

Supplement: Supplementary file 1 — Supporting Information 1 Figure S1: HFHSD leads to obesity and regional comparison of immune cell types in steady‐state. (a) Body weight of mice (n = 15). (b) Intra‐peritoneal glucose tolerance test (IGTT) at indicated time points (n = 15). (c) Lamina propria and intra‐epithelial immune cells of male wild‐type mice at 8 weeks of age as ratios of CD45+ cells (n = 15). Data are pooled from three independent experiments. n represents biologically independent animals. Data are presented as mean ± s.e.m. Two‐sided two‐way repeated measures ANOVA corrected for multiple comparisons (a, b). Two‐way repeated measures ANOVA for tests within subjects (c). ∗ p < 0.05; ∗∗ p < 0.01; ∗∗∗ p < 0.005; ∗∗∗∗ p < 0.001; ns, not significant. [file JIMR-2025-6600076-s004.pdf]

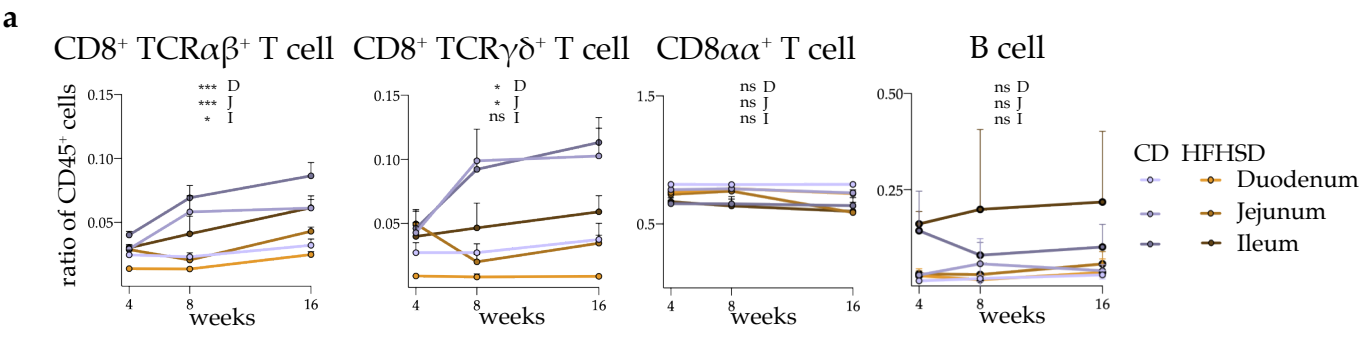

**Figure Supplementary 2**

Supplement: Supplementary file 2 — Supporting Information 2 Figure S2: Effect of diet, location and time on immunophenotype. (a) Ratios of selected lamina propria and intraepithelial immune cell types in CD45+ cells at indicated time points and intestinal segments in HFHSD or CD conditions (n = 15). Data are pooled from three independent experiments. n represents biologically independent animals. Data are presented as mean ± s.e.m (a). [file JIMR-2025-6600076-s003.pdf]

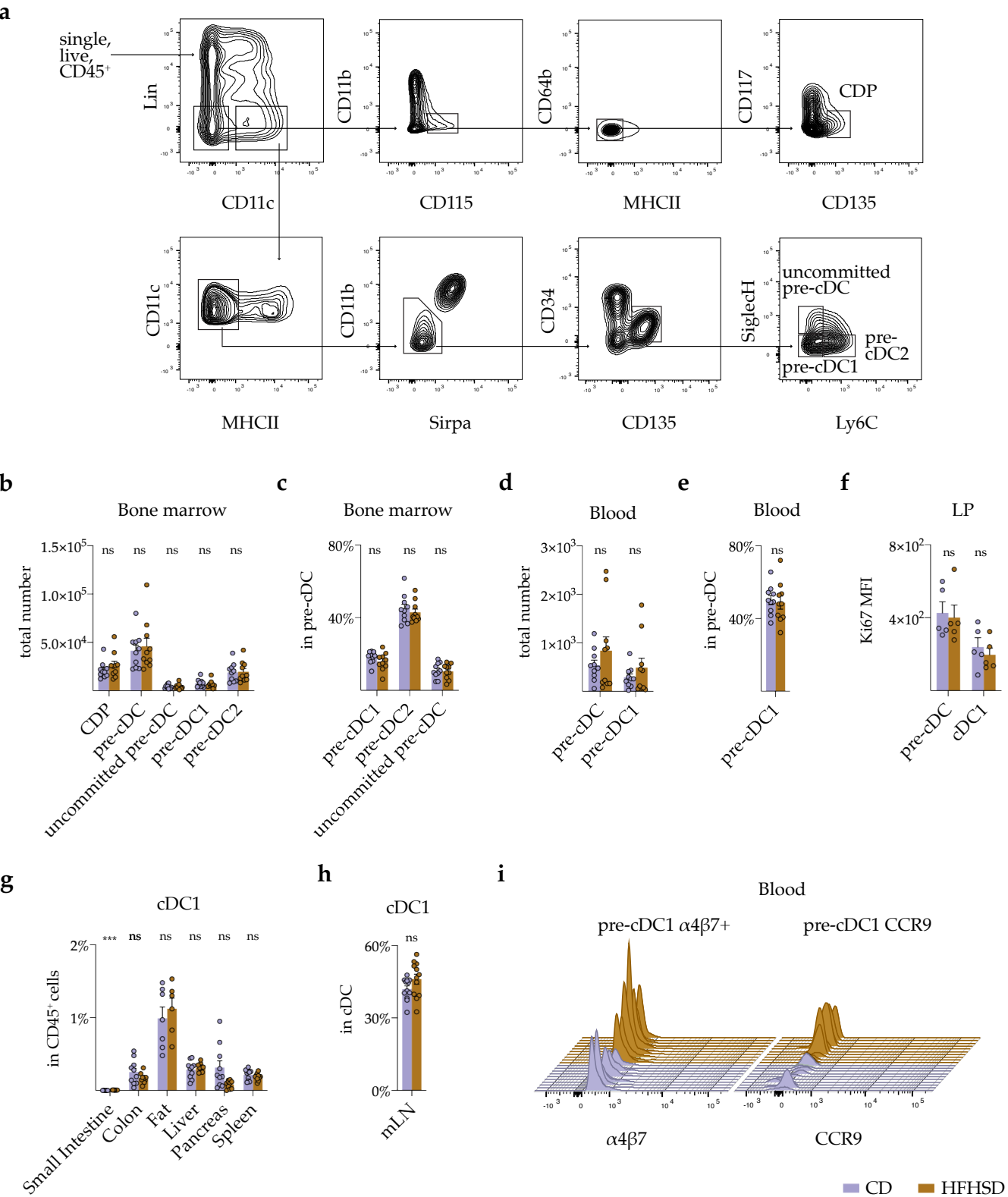

Figure Supplementary 3

Supplement: Supplementary file 3 — Supporting Information 3 Figure S3: Ontogeny and distribution of cDC1 in the obese and steady‐state host. Mice were fed for 8 weeks with HFHSD or CD. (a) Representative gating strategy for identification of cDC precursors by flow cytometry. (b) Total numbers of cDC precursors in bone marrow and (c) as percentage in pre‐cDCs measured by flow cytometry (n = 10). (d) Total numbers of pre‐cDCs and pre‐cDC1 and (e) as percentage of pre‐cDCs in the blood. (f) MFI of Ki67 of cells in the enteric lamina propria (n = 5). (g) cDC1 as percentage in CD45+ cells in metabolically relevant organs and (h) mesenteric lymph nodes (n = 12). (i) Representative histograms showing expression of α4β7 and CCR9 in pre‐DC1 α4β7+ and CCR9+, respectively. Flow cytometry analysis (a–i). Data are pooled from three independent experiments. n represents biologically independent animals. Data are presented as mean ± s.e.m. Two‐tailed unpaired Student’s t‐test (a–g). ∗ p < 0.05; ∗∗ p < 0.01; ∗∗∗ p < 0.005; ∗∗∗∗ p < 0.001. LP, lamina propria; ns, not significant. [file JIMR-2025-6600076-s005.pdf]

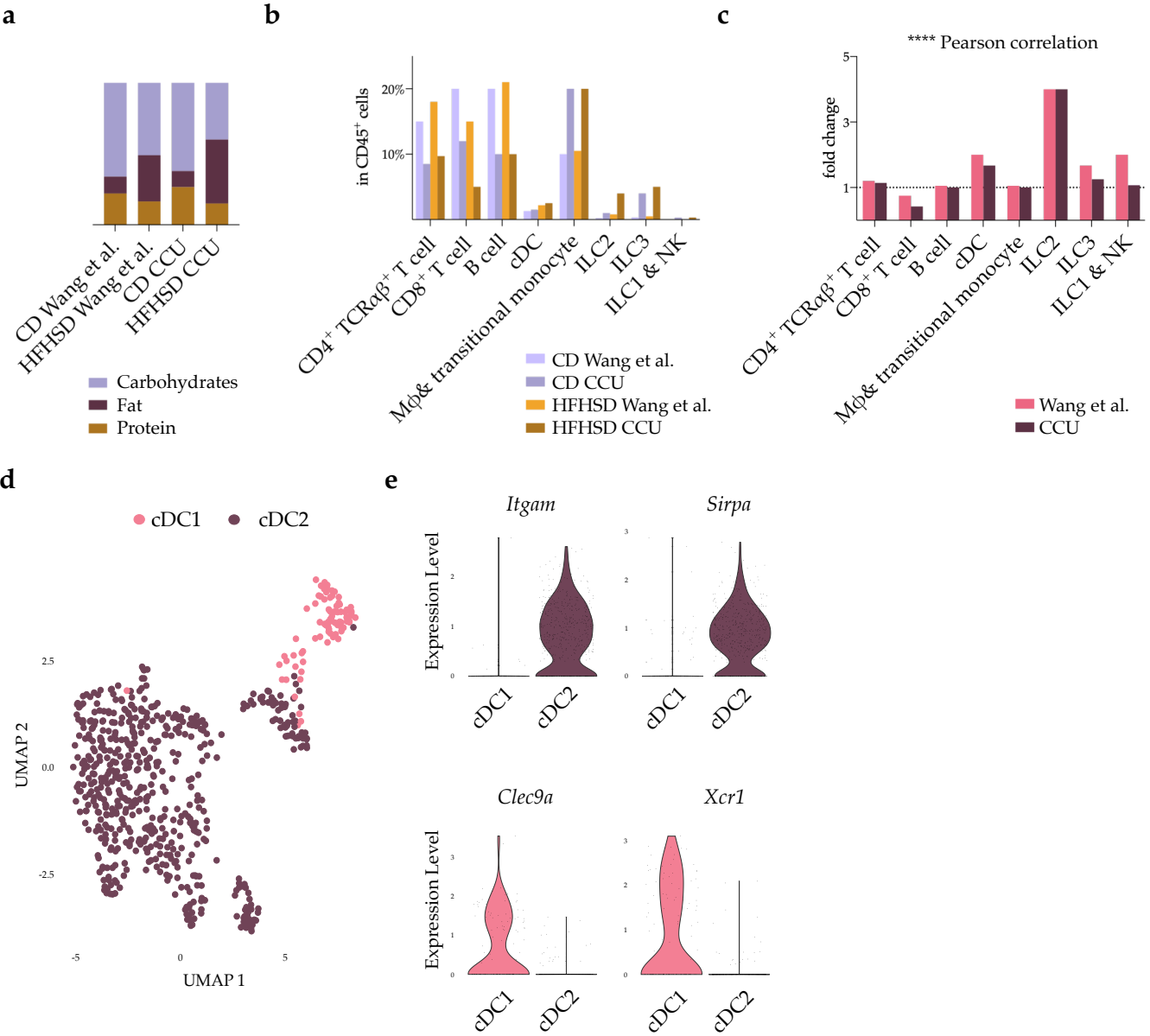

Figure Supplementary 4

Supplement: Supplementary file 4 — Supporting Information 4 Figure S4: Comparison of the scRNA‐seq dataset from Wang et al. [1] and our flow cytometry dataset. (a) Percentage of calories obtained from carbohydrates, fat and proteins in different diets. (b) Side‐by‐side presentation of lamina propria immune cell populations as percentages in CD45+ cells from mice that were fed 8 weeks HFHSD or CD. (c) Fold change of lamina propria immune cell populations from CD to HFHSD. (d) UMAP presentation of cDC subsets in scRNA‐seq dataset from Wang et al. [1]. (e) Marker gene expression used for identification of cDC1 and cDC2 in scRNA‐seq dataset. Pearson correlation (c). DESeq2 analysis (e). CCU, Champalimaud Centre for the Unknown. [file JIMR-2025-6600076-s001.pdf]

**a**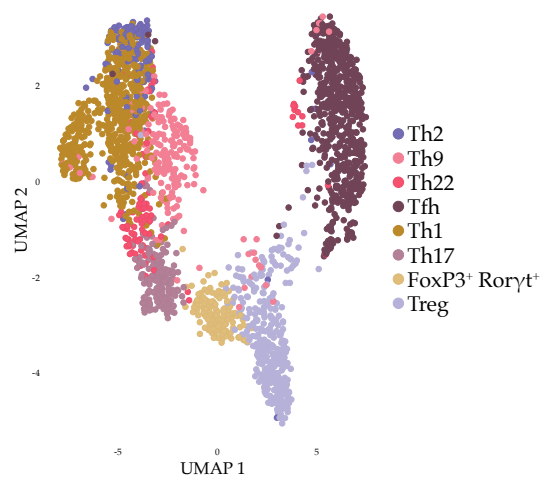**b**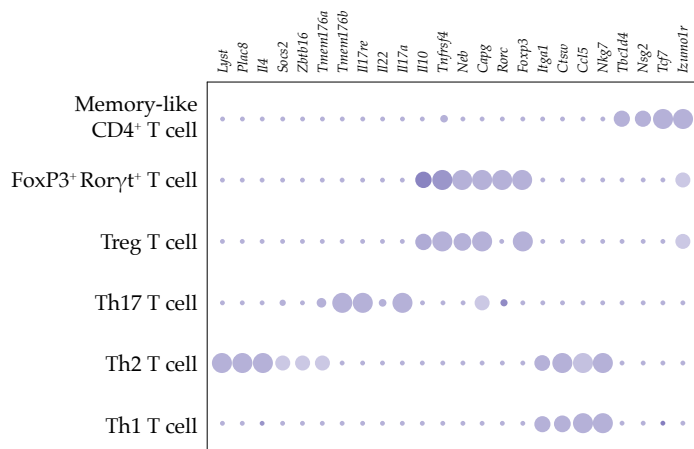

**Figure Supplementary 5**

Supplement: Supplementary file 5 — Supporting Information 5 Figure S5: CD4+ T cell subcluster. (a) UMAP presentation of CD4+ T cell cluster in scRNA‐seq dataset from Wang et al. [1]. (b) Dot plot showing selected top DEG used for identification of CD4+ T cell populations. DESeq2 analysis (b). [file JIMR-2025-6600076-s006.pdf]
